# The Nature of Mental Derangement

**Published:** 1901-04-06

**Authors:** W. Lloyd Andriezen


### Hospital Clinics and Medical Progress.

### THE NATURE OF MENTAL DERANGEMENT.

By W. Lloyd Andriezen, M.D., Lond.

The study of Psychology through the channels of Anatomy, Physiology, Clinical Neurology, Mental Development, Insanity, and Psycho-physical experiments, has gradually served to establish a firm foundation for a Science of Mind. The old assumptions of opposite and irreconcilable metaphysical entities—matter and mind—have given way to a natural and practical biological conception that "mind is a brain function which is found in nearly all animals in varying degrees; which in man arises from small beginnings like any other function, then gradually develops and attains the acme of its complexity and activity in adult life, and finally fails and disappears with the decay of old age." The study of criminology, sociology, and of degeneration, in recent years has served to broaden and strengthen this conception. The fundamental and so-called "organic" instincts of the individual are the feeders of his intellectual, moral, and ethical life. Mental derangement may prevail in the psychical sphere—the domain of intellectual, moral, and ethical life—or may penetrate deeper so as to involve the very foundations of personality. The insanities assume various forms, but fundamentally they are diseases of the brain affecting its psychical functions so as to render the individual dangerous to himself or to others. Hence the need for the sequestration of those who are insane.

The insane have many characteristics in common with the criminal, and it is often hard to draw the line between insanity and criminality. The criminal is often the subject of mental derangement, but this does not of necessity abolish personal responsibility for actions. Even the insane have, and are medically treated as possessors of, a sense of responsibility. In many cases they deserve credit for kind or honest acts and blame for malice and treachery, and in our treatment of them we fully recognise the efficacy of discipline, rewards, and punishments by deprivation of privileges or rewards. An instructive case is quoted by Mercier of an asylum patient, who in the absence of the attendants would bully and attack his more helpless fellow-patients. One day a new patient, a quiet little man, was admitted. The next morning the bully was found in a sorry plight with blackened eyes; he had tried his usual trick on the new patient, who happened to be a prize-fighter by trade, and who administered to the bully a dose which proved a permanent cure.

The nature of mental derangement will be better apprehended when some account is given of the main groups or classes of insanity. In dividing natural objects into classes the biologist has in view not merely convenience of nomenclature and obvious resemblances, but deep-seated affinities and relationships. And so it is with the insanities. The development, course, and termination of the insanities follow certain laws which have been extensively observed and minutely studied in the past decade. The chief law of the pathogenesis of the insanities may be formulated thus: "Of the various evolutions in brain and mind, from the fœtal to the sexually mature stage, modifications may occur in the form of defect, arrest, or morbid alteration, and such pathological conditions are the bases of the insanities."

The insanities thus regarded are seen to constitute groups which assume a serial or tree-like arrangement. At the lower end of the series is situated the group marked by profound arrests of cerebral and psychical development, with absence or deficiency of evolution of personality. This constitutes the group of idiots. Two main classes are recognised here, namely, (*a*) idiots of vegetative and somnolescent grade, *e.g.*, many paralytic, hydrocephalic, and congenital idiots, of wet and dirty habits, helpless, and ineducable. The second class consists of (*b*) idiots of medium and higher grade, slightly improvable and educable. They comprise many microcephalic, cretinoid, epileptic, partially paralytic, or simple genetic idiots.

Above the group of idiots is placed the group of imbeciles. These occupy a higher position in the psychological scale. They are characterised by a feebleness or insufficiency of cerebral and psychical development, with a parallel enfeeblement in the development of intellectual, moral, and social activities. They may be arranged in four grades as follows: (*a*) imbeciles of low grade, not educable; (*b*) imbeciles of medium grade, partially educable and improvable; (*c*) imbeciles of higher grade, partially educable and improvable, but often possessed of anti-social instincts; and (*d*) feeble-minded persons, who are partially educable and improvable and who, though often possessed of anti-social instincts, are more amenable to moral improvement and industrial training.

Above the group of imbeciles is placed a vast and mixed host comprising various types of mental degeneracy—a group to which I have elsewhere applied the title of paraphrenia. The paraphreniacs comprise a large number and variety of classes, *e.g.*, cranks, eccentrics, mattoids, mystics prone to hallucinations, dipsomaniacs, kleptomaniacs, cases of agoraphobia and of *folie du doute*, psychopates of erotic and litigious types, congenital sexual perverts and moral perverts, congenital criminals of both the active and the neurasthenic types, paranoiacs, the subjects of cyclic insanity (*folie circulaire*), and those tainted with a hereditary neurotic anomaly in the form of hysterical insanity, epileptic insanity, choreic insanity, and dementia praxcox.

The insanities which occur in brains of nearly full psychical development and of apparent previous health form the next group—the phrenopathies. Several classes belong to this group. Thus we have (*a*) the vesanic type (*e.g.* melancholia, mania, stupor, acute mental confusion), (*b*) the toxic type (*e.g.* the insanities immediately due to alcoholism, morphinism, cocainism, lead poisoning, and ergotism), (*c*) the microparasitic febrile or post-febrile type (*e.g.* some of the puerperal insanities, acute delirium, the psychoses of influenzal and scarlatinal origin, and those following tubercular, epidemic cerebro-spinal and other forms of meningitis), (*d*) the diathetic group associated with serious derangements of general bodily metabolism (*e.g.* the insanities of myxœdema, goitre, acromegaly, and possibly diabetes and syphilis), (*e*) the insanities of chronic meningo-encephalitis, whether of para-syphilitic or other origin (*e.g.* general paralysis of the insane), (*f*) the insanities associated with involutional changes of middle and old age (*e.g.* climacteric insanity, chronic cerebral atrophy, and senile insanity), (*g*) traumatic insanity, and (*h*) insanity from gross organic lesions (*e.g.* cerebral neoplasms, thrombosis, and hæmorrhages).

Finally comes the group which includes the wrecks and remnants of previous attacks of insanity, chronic derelicts deprived of their higher mental equipments and reduced to lower grades and levels of mental life—a highly artificial, motley, and lumber-room group, the chronic and secondary dements—who might for convenience and uniformity of nomenclature be called lipophreniacs. These five main groups include the various known types of mental disease.

The inheritance of disease is no greater mystery than that of health. The normal child is born with a life destiny, an endowment of capacities and latent tendencies which will make him at first a feeder and animal parasite (fœtal and infantile stage), next a sensitive but irrational being (early childhood), next a rational being gradually developing a sexual life (pre-adolescent and adolescent stage), next a citizen with a place in the social fabric and destined to take his part in the struggle of life (adult life), and finally subject to the weakening of function which inevitably follows middle-life and which ushers in the decay of old age. Inherited tendencies to mental derangement may appear at any one of these periods of life, the various epochs of life having their special pathological tendencies as well as normal developments. What is inherited is, in most cases, the morbid characteristics which constitute a tendency to this or that generic type of disease. The influences of the environment will then determine the particular form in which the disease may app ar Marriage may modify (diminish or augment) the hereditary transmission of such tendencies according as the crossing is with healthy or tainted blood. Finally the special epoch of life in which an insanity may develop will also contribute its "local colouring" to the kind of insanity in question.

